# Impact of air pollution on physician office visits for common childhood conditions in Ontario, Canada

**DOI:** 10.1186/1710-1492-10-S2-A54

**Published:** 2014-12-18

**Authors:** Laura Feldman, Chenwei Gao, Jingqin Zhu, Jacqueline Simatovic, Teresa To

**Affiliations:** 1 Child Health Evaluative Sciences, The Hospital for Sick Children, Toronto, Ontario, M5G 1X8, Canada; 2 University of Toronto, Toronto, Ontario, M5S 1A1, Canada; 3 Institute for Clinical Evaluative Sciences, North York, Ontario, M5T 3M6, Canada

## Background

Children are particularly sensitive to air pollutants, due to factors such as ongoing lung development and choice of activities [1]. We evaluated the impact of fine particulate matter (PM_2.5_) on physician office visits for common conditions in children in Ontario, Canada.

## Methods

PM_2.5_ and temperature measurements were obtained from satellite data for all of Ontario [2]. Physician office visits were stratified into two groups based on the literature: air pollution-sensitive (acute respiratory infections, allergic rhinitis, asthma, bronchiolitis, diabetes, otitis media) and air pollution-insensitive (gastroenteritis, injuries). Claims data were obtained for every month in 2010 from health administrative databases for children 0-14 years of age. Age- and sex-standardized morbidity ratios (SMRs) were calculated by region in Ontario. Spatial Poisson regression models were used to analyze the relationship between PM_2.5_ and physician office visits, with temperature as a covariate.

## Results

**Figure 1:**
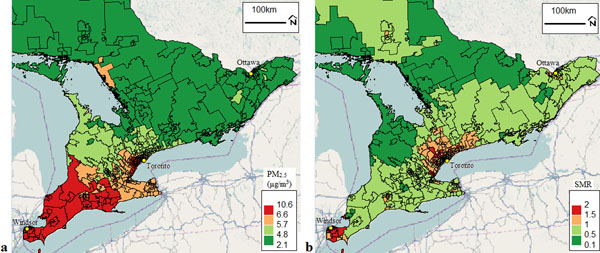
Distribution of (a) fine particulate matter (PM_2.5_, in μg/m^3^) and (b) fitted sex-standardized morbidity ratios (SMRs) from spatial Poisson regression for physician office visits for air pollution-sensitive conditions; all by Forward Sortation Area (FSA) in Southern Ontario in July 2010

Crude rates of physician office visits are presented in Table 1. As expected, fine particulate was significantly associated with monthly rates of physician office visits for air pollution-sensitive conditions, and not for insensitive conditions. Fitted SMRs for air pollution-sensitive conditions are presented in Figure 1. SMRs for sensitive and insensitive conditions were strongly positively correlated (r = 0.53), and data were spatially autocorrelated. This suggests an underlying spatial process that influences physician office visit rates for common childhood conditions, both for air pollution-sensitive and -insensitive conditions.

**Table 1:** Crude rates of air pollution-sensitive and air pollution-insensitive conditions in Ontario for each month in 2010

	Crude rates of physician office visits^a^											
	Jan	Feb	Mar	Apr	May	June	July	Aug	Sept	Oct	Nov	Dec

Air pollution-sensitive	8.05	8.84	8.94	7.80	7.21	6.55	5.15	4.73	6.57	7.52	9.27	10.89

Air pollution-insensitive	1.48	1.52	1.61	1.55	1.63	1.63	1.34	1.29	1.34	1.38	1.54	1.22

^a^Number of claims per 100 population aged 0-14 years.

## Conclusions

In this analysis PM_2.5_, was significantly associated with physician office visits for air pollution-sensitive conditions. Areas with high PM2.5 levels and SMRs higher than 1 were identified; children with air pollution-sensitive conditions in these areas may benefit from targeted air pollution reduction interventions. Additionally, future analysis should evaluate the role of household income and access to care in influencing the spatial pattern of primary health care utilization for common childhood conditions across Ontario.
